# Supplementary material for: Transparent Packaging for Tea: Exploring the Role of Trust in Emerging Markets for Tea Beverages
Source: Foods. 2025 Nov 14;14(22):3893. doi: 10.3390/foods14223893 (PMC12651621; doi:10.3390/foods14223893)
Supplement: Supplementary file 1 [file foods-14-03893-s001.zip › foods-3953169-supplementary.pdf]

Table S1.

Measures.

Constructs	Items	Scale	Reliability	References
Purchase Intention	1. How much do you want to purchase this tea beverage?	1= Extremely low<br>5 = Extremely high	0.95 (Study 1)	Konuk (2019)
	2. How likely would you be to purchase this tea beverage?			
Perceived Trust	1. I believe the quality of this tea beverage is reliable.	1 = Strongly disagree<br>5 = Strongly agree	0.91 (Study 2)<br>0.78 (Study 3)	Kim and Song (2020)
	2. I believe the quality of this tea beverage is assured.			
Perceived healthiness	1. I believe this tea beverage is healthy.	1 = Strongly disagree<br>5 = Strongly agree	0.97 (Study 1)<br>0.91 (Study 2)<br>0.81 (Study 3)	Yamim et al. (2020)
	2. I believe this tea beverage is beneficial for my health.			
Perceived tastiness	1. I believe this tea beverage is delicious.	1 = Strongly disagree<br>5 = Strongly agree	0.92 (Study 1)<br>0.89 (Study 2)<br>0.81 (Study 3)	Yamim et al. (2020)
	2. I believe this tea beverage is tasty.			

Note:
a. The demographic measures include gender, age, education level, the frequency of purchasing tea beverages, and monthly disposable income.
b. In Studies 2 and 3, participants indicated their purchase intention by ranking the tea beverages, with the top-ranked option representing the one they were most willing to purchase.

References:

Kim, J.-H., & Song, H. (2020). The influence of perceived credibility on purchase intention via competence and authenticity. *International Journal of Hospitality Management, 90*, 102617. doi:10.1016/j.ijhm.2020.102617

Konuk, F. A. (2019). Consumers' willingness to buy and willingness to pay for fair trade food: The influence of consciousness for fair consumption, environmental concern, trust and innovativeness. *Food Research International, 120*, 141-147. doi:10.1016/j.foodres.2019.02.018

Yamim, A. P., Mai, R., & Werle, C. O. (2020). Make it hot? How food temperature (mis) guides product judgments. *Journal of Consumer Research, 47*(4), 523-543. doi:10.1093/jcr/ucaa017

Table S2.

The research materials used in Study 1.

Transparent packaging	Opaque packaging		
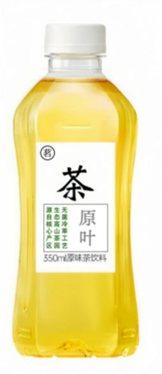	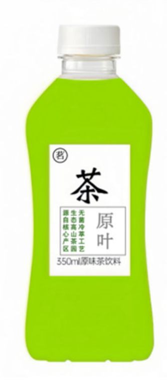	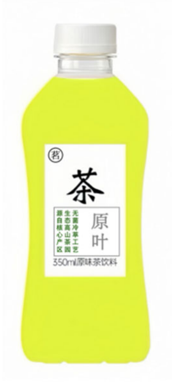	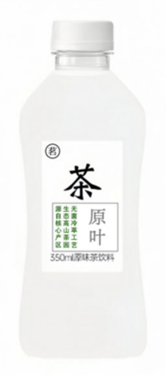

Table S3.

The research materials used in Study 2.

Transparent packaging	Opaque red packaging	Opaque green packaging
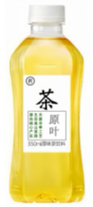	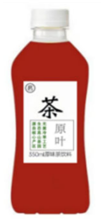	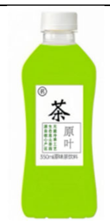

Note. The positions were counterbalanced.
